# The function of plant PR1 and other members of the CAP protein superfamily in plant–pathogen interactions

**DOI:** 10.1111/mpp.13320

**Published:** 2023-03-17

**Authors:** Zhu Han, Dianguang Xiong, Roger Schneiter, Chengming Tian

**Affiliations:** ^1^ College of Forestry Beijing Forestry University Beijing China; ^2^ Department of Biology University of Fribourg Fribourg Switzerland

**Keywords:** effector proteins, fungal CAPs, nematode VALs/VAPs, pathogen virulence, plant immunity, plant PR1, sperm‐coating proteins (SCPs)

## Abstract

The pathogenesis‐related (PR) proteins of plants have originally been identified as proteins that are strongly induced upon biotic and abiotic stress. These proteins fall into 17 distinct classes (PR1–PR17). The mode of action of most of these PR proteins has been well characterized, except for PR1, which belongs to a widespread superfamily of proteins that share a common CAP domain. Proteins of this family are not only expressed in plants but also in humans and in many different pathogens, including phytopathogenic nematodes and fungi. These proteins are associated with a diverse range of physiological functions. However, their precise mode of action has remained elusive. The importance of these proteins in immune defence is illustrated by the fact that PR1 overexpression in plants results in increased resistance against pathogens. However, PR1‐like CAP proteins are also produced by pathogens and deletion of these genes results in reduced virulence, suggesting that CAP proteins can exert both defensive and offensive functions. Recent progress has revealed that plant PR1 is proteolytically cleaved to release a C‐terminal CAPE1 peptide, which is sufficient to activate an immune response. The release of this signalling peptide is blocked by pathogenic effectors to evade immune defence. Moreover, plant PR1 forms complexes with other PR family members, including PR5, also known as thaumatin, and PR14, a lipid transfer protein, to enhance the host's immune response. Here, we discuss possible functions of PR1 proteins and their interactors, particularly in light of the fact that these proteins can bind lipids, which have important immune signalling functions.

## Graphical abstract

Secreted pathogenesis‐related 1 (PR1) proteins are highly conserved and strongly induced upon stress. Phytopathogens require PR1‐like proteins for virulence, suggesting competition of these proteins in the extracellular space.

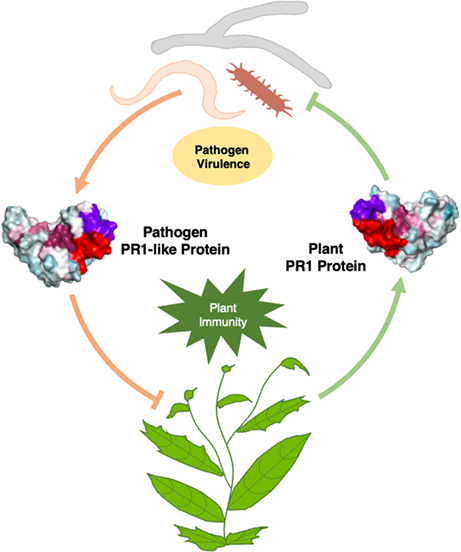

## 1 OVERVIEW OF CAP SUPERFAMILY PROTEINS

CAP proteins constitute an evolutionarily conserved and widely expressed protein superfamily with members across the bacterial, fungal, plant, and animal kingdoms. This protein family was named after its three founding members: the cysteine‐rich secretory proteins from humans (CRISP), antigen 5 (Ag5) from stinging insects, and pathogenesis‐related 1 (PR1) proteins from plants. These proteins are also known as sperm‐coating proteins (SCPs), or venom allergen‐like proteins (VALs or VAPs). Most CAP proteins are secreted glycoproteins and they harbour a 17–21‐kDa globular CAP domain, which adopts a unique αβα sandwich fold. The structural conservation of the CAP domain and the fact that most family members are secreted suggests that these proteins share a common function within the extracellular space, but their precise mode of action remains to be defined (Cantacessi & Gasser, 2012; Gibbs et al., 2008; Wilbers et al., 2018).

Over the past decades, insights into the many diverse functions of these proteins in complex physiological processes such as fertilization, the host immune response, pathogen virulence, venom toxicology, and cancer progression have been gained (Gaikwad et al., 2020; Schneiter & Di Pietro, 2013; Tadokoro et al., 2020). These proteins have even been explored as potential vaccine candidates against parasitic gastrointestinal infections caused by hookworm nematodes (Darwiche et al., 2018; Mendez et al., 2008).

In this article, we review the current state of our understanding of the function of CAP superfamily proteins from plants and their fungal and nematode pathogens. We will highlight the structural conservation of the CAP domain and emphasize the role of CAP superfamily proteins in plant–pathogen interactions. Their function in host–pathogen interactions is particularly intriguing as both the hosts and the pathogens strongly induce expression of their respective CAP proteins during different stages of interactions, suggesting a cross‐kingdom tug‐of‐war type of action of these proteins within the extracellular space. While plant PR1 proteins are part of the host immune response, PR1‐like proteins produced by the pathogens are important for their virulence by suppressing the host's immune responses and promoting colonization. Here, we will provide insights into a possible common function of these proteins in binding lipids and related small hydrophobic compounds to integrate lipid binding with immune signalling.

## 2 CAP PROTEINS ADOPT A UNIQUE CONSERVED SANDWICH FOLD WITH A LARGE CENTRAL CAVITY

**FIGURE 1:**
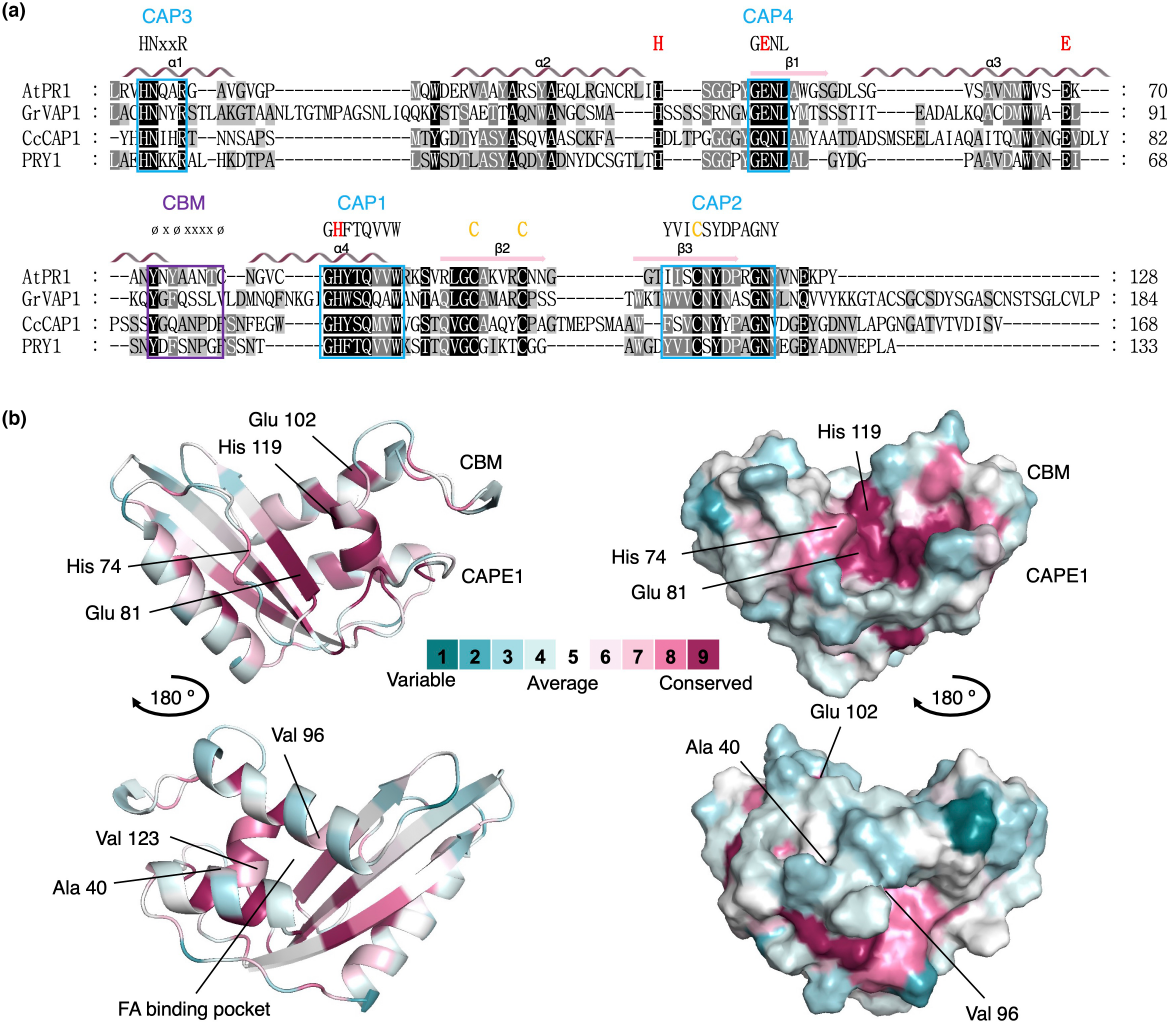
Structural conservation of the CAP domain. (a) Sequence alignment of CAP family members from *Arabidopsis thaliana* (AtPR1), from the potato cyst nematode *Globodera rostochiensis* (GrVAP1), from the phytopathogenic fungus *Cytospora chrysosperma* (CcCAP1), and from the yeast *Saccharomyces cerevisiae* (PRY1). The CAP1–4 signature motifs are boxed in blue. The highly conserved histidine and glutamic acid residues that constitute the conserved tetrad residues are indicated in red. The four alpha‐helices (α1–α4) are shown as wavy lines. The conserved aromatic amino acids (ØXØXXXXØ) that define the caveolin‐binding motif (CBM) in the flexible loop that connects helix α3 with α4 is highlighted in a purple box. The three cysteines that are highly conserved in all four proteins are indicated in orange. (b) Tertiary structure of the CAP domain from AtPR1. Conserved elements as determined by ConSurf (Ashkenazy et al., 2016) are indicated in red and the highly conserved histidine and glutamic acid residues are indicated, as is the CBM, the CAPE1 motif, and the fatty acid (FA) binding pocket formed by helices α1, α3, and α4. Key residues Ala40, Val96, and Val123 in the FA binding pocket are indicated. The structure is represented by a ribbon diagram and as a space‐filling model.

CAP proteins share limited sequence identity with each other and are defined by the presence of four conserved CAP signature motifs (CAP1–4) (Gibbs et al., 2008) (Figure 1). At the structural level, CAP proteins adopt a unique compact three‐layered αβα sandwich fold harbouring a central CAP cavity. This cavity is large enough to potentially bind peptides or carbohydrates (Shoji‐Kawata et al., 2013). The cavity is lined by four highly conserved, partially surface‐exposed residues, two histidine and two glutamic acid residues. These CAP tetrad residues coordinate divalent cations including Zn^2+^ and Mg^2+^ (Asojo et al., 2011; Darwiche et al., 2016; Milne et al., 2003; Szyperski et al., 1998; Wang et al., 2010). The CAP domain is stabilized by hydrophobic interactions, multiple hydrogen bonds, and two highly conserved disulphide bonds. These interactions are thought to provide the thermal, pH, and proteolytic stability required for the extracellular function of these proteins (Fernandez et al., 1997; Szyperski et al., 1998). The importance of the two disulphide bridges is illustrated by the observation that two of the four CAP proteins from the wheat head blight fungus *Fusarium graminearum*, FgPR1L‐3 and FgPR1L‐4, that have lost these cysteine residues are hypersensitive to proteolysis (Lu & Edwards, 2018).

CAP domains differ from each other mostly in the length and orientation of the loops, which connect the highly conserved secondary structure elements that make up the sandwich fold (Asojo et al., 2011; Baroni et al., 2017; Darwiche et al., 2016). One of these loops harbours a so‐called caveolin‐binding motif (CBM), which is defined by the presence of aromatic amino acids in positions 1, 3, and 8 of the motif (ØXØXXXXØ) (Couet et al., 1997; Eberle et al., 2002). The flexibility of this loop and the presence of aromatic residues in this motif are important for the ability of CAP proteins to bind sterols and related small hydrophobic compounds (Choudhary et al., 2014; Cottier et al., 2020; Darwiche, El Atab, et al., 2017; El Atab et al., 2022). Based on the fold of a nuclease A inhibitor, which bears a rare variation of the sandwich fold, the flexible loop that harbours the CBM was previously proposed to be of functional importance to CAP proteins (Kirby et al., 2002). It was proposed that the purpose of the sandwich fold is to form a stable scaffold to present this extended loop structure for biological interactions with other proteins or small ligands (Kirby et al., 2002). The CBM motif is mostly preserved in CAP proteins of mammals, flies, worms, and fungi, but not in organisms such as plants, wasps, ants, or snakes (Choudhary et al., 2014).

Structural analysis of tablysin‐15 from the saliva of the blood‐feeding horsefly *Tabanus yao* revealed a second lipid‐binding site in the CAP domain, consisting of a hydrophobic channel formed by helices α1, α3, and α4 (Xu et al., 2012) (Figure 1). This channel binds proinflammatory leukotrienes with submicromolar affinity but can also bind long‐chain fatty acids (Xu et al., 2012). Tablysin‐15 thereby functions as a scavenger of eicosanoids to inhibit integrin function, platelet activation, and blood coagulation upon insect feeding. The sterol‐ and fatty acid‐binding sites within the CAP domain are non‐overlapping and function independently of each other, suggesting that the CAP domain serves as a stable, secreted protein domain that can accommodate multiple ligand‐binding sites (Darwiche, Mène‐Saffrané, et al., 2017).

Most CAP proteins contain a single evolutionary ancient CAP domain (Abraham & Chandler, 2017). However, some of these single‐CAP domain proteins can form homodimers, as shown for TaPR1‐5 from wheat (*Triticum aestivum*), Fpr1 from the soilborne fungal pathogen *Fusarium oxysporum*, and Pry1 from the yeast *Saccharomyces cerevisiae* (Darwiche et al., 2016; Lu et al., 2013; Prados‐Rosales et al., 2012). Other CAP family members harbour tandem CAP domains as first revealed by structural characterization of Na‐ASP‐1, a CAP protein from the human hookworm parasite *Necator americanus* (Asojo, 2011; Lu et al., 2017). Finally, some proteins can even contain multiple CAP domains, with up to eight covalently linked CAP domains present in an uncharacterized protein from the freshwater microalga *Raphidocelis subcapitata* (A0A2V0P999_9CHLO). This type of concatenation of CAP domains within a single protein suggests that the domain exerts a stoichiometric rather than catalytic function (http://pfam.xfam.org/family/CAP).

The CAP domain is often linked to other functional domains as N‐ and/or C‐terminal extensions. These flanking domains have been proposed to modulate the function of the CAP domain, possibly to affect target specificity and thereby control the precise nature of the physiological responses (Gibbs et al., 2008). For example, transmembrane and protein kinase domains have been identified in CAP proteins from cacao, wheat, rice, and several other plant species, suggesting that the CAP domain of these family members are directly coupled to an intracellular signal transduction pathway (Breen et al., 2017; Lu et al., 2017; Teixeira et al., 2013).

## 3 CAP PROTEINS FROM PHYTOPATHOGENIC FUNGI AND NEMATODES ARE IMPORTANT FOR VIRULENCE AND SUPPRESSION OF THE HOST'S IMMUNE RESPONSE

Though CAP proteins are being studied in many diverse species, pathogens may provide the most fascinating insight into the function of these proteins, as they participate in promoting virulence and suppressing the immune response of the host. In addition, uncovering the mode of action of these proteins in host–pathogen interactions may allow for an entry point to reduce virulence of pathogens, which pose a threat to agriculture and forestry.

### 3.1 CAP proteins of plant‐pathogenic fungi

Over the last couple of years, CAP proteins from different pathogenic fungi have emerged as novel virulence factors. For example, in the hemibiotrophic *Fusarium* species, the CAP proteins Fpr1 from the ubiquitous pathogen *F. oxysporum*, its homologue from the wheat head blight fungus *F. graminearum* (FgPR1L‐4), and FvSCP1 from the maize pathogen *Fusarium verticillioides* have all been shown to promote fungal virulence during host infection (Lu & Edwards, 2018; Prados‐Rosales et al., 2012; Zhang, Mukherjee, et al., 2018) (Table 1). Importantly, the virulence activity of these CAP proteins requires the highly conserved CAP tetrad residues, as *Fusarium* strains expressing a mutant version of Fpr1 lose virulence when tested in a disseminated immune‐depressed mouse model (Prados‐Rosales et al., 2012).

**TABLE 1:** Overview of CAP/VAL/VAP proteins from phytopathogenic fungi and nematodes.

Phytopathogen		Host plant	CAP proteins	Virulence^a^	Gene ID	GPI/TMD	References
Necrotrophic fungi	*Cytospora* *chrysosperma*	Poplar	CcCAP1	Yes	GME7477_g		Han et al. (2021)
			CcCAP2	Yes	GME9352_g		
			CcCAP3	No	GME10144_g	GPI	
	*Valsa* *mali*	Apple	VmPR1a	Yes			Meng et al. (2019); Wang et al. (2021)
			VmPR1b	No			
			VmPR1c	Yes			
Hemibiotrophic fungi	*Moniliophthora* *perniciosa*	Cacao	MpPR1a	nd	JN620340		Teixeira et al. (2012); Baroni et al. (2017); Darwiche, El Atab, et al. (2017)
			MpPR1b		JN620341		
			MpPR1c		JN620342		
			MpPR1d		JN620343		
			MpPR1e		JN620344		
			MpPR1f		JN620345		
			MpPR1g		JN620346		
			MpPR1h		JN620347	TMD	
			MpPR1i		JN620348		
			MpPR1j		JN620349		
			MpPR1k		JN620350		
	*Fusarium* *graminearum*	Wheat	FgPR1L‐1	No	FGSG_00569		Lu and Edwards (2018)
			FgPR1L‐2	No	FGSG_02744	GPI	
			FgPR1L‐3	No	FGSG_03109		
			FgPR1L‐4	Yes	FGSG_03312		
	*Fusarium* *verticillioides*	Maize	FvSCP1	Yes	FVEG_04097		Zhang, Mukherjee, et al. (2018)
					FVEG_08950	GPI	
					FVEG_10833		
					FVEG_08686		
	*Fusarium* *oxysporum* f. sp. *lycopersici*	Tomato	Fpr1	No	FOXG_09795		Prado‐Rosales et al. (2012)
					FOXG_06245		
					FOXG_10300		
					FOXG_14109		
					FOXG_12292		
Nematode	*Globodera rostochiensis*	Tomato	GrVAP1	Yes	AW506232	TMD	Lozano‐Torres et al. (2012, 2014)
	*Heterodera avenae*	Barley	HaVAP1	Yes	MH255798	TMD	Luo et al. (2019)
			HaVAP2	Yes	MH255799	TMD	
	*Radopholus similis*	Tomato	RsVAP	Yes			Li et al. (2021)
	*Meloidogyne hispanica*	Tomato	MhiVAP1	Yes	MhA1_Contig2874		Duarte et al. (2017)

Abbreviations: GPI, glycosylphosphatidylinositol; TMD, transmembrane domain.

^a^ Virulence indicates whether deletion of the respective gene reduces pathogen virulence. nd, not determined.

One of the largest families of CAP proteins in fungal pathogens, containing 11 members, has been identified in the genome of *Moniliophthora perniciosa*, a commercially important and devastating agent causing witches' broom disease in cacao plants. Most of the *M. perniciosa* CAP proteins are highly and specifically induced during the course of infection (Teixeira et al., 2012). Some of these proteins contain a functional CBM and have been shown to bind sterols in vitro and in a yeast‐based in vivo sterol export assay (Darwiche, El Atab, et al., 2017).

The necrotrophic pathogens *Cytospora chrysosperma* and *Valsa mali*, the causative agents of poplar and apple canker disease, both harbour three related CAP proteins. Deletion of *C. chrysosperma* CcCAP1 does not affect vegetative growth but decreases fungal virulence and renders the mutant fungal cells sensitive to reactive oxygen species (ROS) (Han et al., 2021). Similarly, two of the three *V. mali* CAP proteins, VmPR1a and VmPR1c, are important for pathogen virulence (Wang et al., 2021). Interestingly, the three CAP proteins present in these two tree pathogens share a high (>73%) sequence similarity with each other. However, deletion of the third CAP family member in either *C. chrysosperma* (CcCAP3) or *V. mali* (VmPR1b) does not affect their virulence, suggesting that these proteins exert additional functions that are not readily assessed by virulence assays (Han et al., 2021; our unpublished observations). After secretion from the pathogen, both CcCAP1 and VmPR1c appear to translocate into the cytoplasm and nucleus of their host cells to interfere with the plant's immune response. While CcCAP1 inhibits plant pathogen‐associated molecular pattern (PAMP)‐triggered immunity (PTI) and promotes fungal colonization, VmPR1c induces PR1 expression in the host and cell death (Han et al., 2021).

### 3.2 CAP/VAL/VAP proteins of plant‐pathogenic nematodes

Phytonematodes pose serious threats to plants, causing a worldwide loss of roughly 10% of food crop yields per year. When infecting plants, parasitic nematodes secrete small organic compounds and proteins through their stylet and body wall into the apoplast and cytoplasm of host cells to induce the formation of permanent feeding structures (Desmedt et al., 2020; Zheng et al., 2021). These form the sole source of plant nutrients for sedentary nematodes. Apart from altering host cell metabolism and function, nematodes secrete effectors to modulate host immunity (Smant et al., 2018). These proteins are produced by the oesophageal glands and include CAP proteins, which in the context of pathogenic nematodes are frequently referred to as VAL/VAP proteins. They are not restricted to plant‐pathogenic nematodes and constitute some of the most abundantly secreted proteins during the onset of parasitism in some animal‐parasitic nematodes (Cantacessi & Gasser, 2012; Wilbers et al., 2018). The abundance and unique conservation of VAL/VAP proteins in the secretion of parasitic nematodes might point to a common activity of these effector proteins within the extracellular matrix of both animal and plant cells.

During colonization of their host, nematodes deposit VAL/VAP proteins into the apoplastic space of their host plants (Lozano‐Torres et al., 2014; Luo et al., 2019). The expression of these VAL/VAP genes coincides with stages of nematode invasion and migration through the host tissues (Lozano‐Torres et al., 2012). These CAP proteins play a significant role in aggressiveness of nematodes, as illustrated by MhiVAP1, produced by the important root‐knot nematode *Meloidogyne hispanica*. RNA interference‐mediated silencing of MhiVAP1 greatly interferes with the completion of the nematode life cycle and attenuates its attraction to roots, penetration, and colonization of tomato plants, indicating its importance during early stages of infection (Duarte et al., 2017).

Following the discovery of a virulence‐related VAL/VAP protein in the potato cyst nematode *Globodera rostochiensis*, investigations focused on the function of this GrVAP1 protein in the suppression of the host immune response. Expression of GrVAP1 in plants results in loss of basal immunity to multiple unrelated pathogens (Lozano‐Torres et al., 2014). To suppress immunity, GrVAP1 targets the extracellular papain‐like cysteine protease Rcr3 of tomato (Lozano‐Torres et al., 2014). Tomato variants that are resistant to *G. rostochiensis*, on the other hand, trigger a defence‐related response that is mediated by the surface‐localized immune receptor Cf‐2 (Lozano‐Torres et al., 2012). Cf‐2 senses GrVAP1 indirectly, through detection of perturbations of its co‐receptor Rcr3, resulting in the activation of resistance and the induction of a localized programmed cell death response, known as the hypersensitive response (HR) (Heath, 2000; Lozano‐Torres et al., 2012).

The mechanism underlying the Rcr3‐mediated defence response was revealed by a recent study. Generation of the functional, mature form of Rcr3, mRcr3, requires the processing of its catalytic inactive precursor, proRcr3, by a group of subtilisin‐like serine proteases, subtilases, which thereby generate a binding site for the effector GrVAP1 on mRcr3 (Paulus et al., 2020). Because distantly related subtilases can also activate proRcr3, these observations might point to a network of proteolytic cascades in solanaceous plants that control apoplastic immunity (Kourelis et al., 2020; Paulus et al., 2020). Rcr3 is targeted not only by GrVAP1, but also by the avirulence factor Avr2 from the leaf mould fungus *Cladosporium fulvum*, by the bacterial protease inhibitor Cip1, and by several oomycete effectors (Ilyas et al., 2015; Misas Villamil et al., 2019; Rooney et al., 2005; Zheng et al., 2021). Rcr3 and related papain‐like immune proteases thus appear to serve as common targets for independently evolved virulence factors from different pathogens, including CAP/VAL/VAP family members, indicating that Rcr3 serves as an important component of the extracellular immune system.

The VAL/VAP proteins from the cereal cyst nematode *Heterodera avenae*, HaVAP1 and HaVAP2, are both induced during the parasitic juvenile stages, contribute to the onset of parasitism, and inhibit BAX‐induced host cell death (Luo et al., 2019). Similarly, the VAL/VAP protein RsVAP, from the important migratory root endoparasitic nematode *Radopholus similis*, is highly induced in juveniles and its silencing decreases pathogenicity on tomato. On the other hand, overexpression of RsVAP in tobacco leaves inhibits a PTI response triggered by a bacterial flagellin‐derived peptide (flg22) and prevents a cell death response, suggesting that RsVAP represses the plant's basal immunity (Li et al., 2021).

Taken together, the observations that VAL/VAP proteins are highly expressed in the oesophageal glands of plant‐pathogenic nematodes and injected into the apoplastic space during invasion suggest that these proteins function as apoplastic suppressors of an immune response triggered, for example, by plant cell wall fragments that are released during nematode migration inside the host plants (Tanaka & Heil, 2021). A better characterization of a possibly common mode of action of VAL/VAPs might help to define target genes for the future development of novel nematicidal strategies.

## 4 PLANTS RECOGNIZE PATHOGENS AND MOUNT AN IMMUNE RESPONSE

Terrestrial plants protect themselves with two lines of immune defence. The first, a passive defence, serves to prevent pathogen invasion through cutin, waxes, rigid lignin, and callose depositions on the cell wall, and is combined with a chemical barrier, which includes various cytostatic and antimicrobial phytochemicals (Saur & Hückelhoven, 2021). The second is an inducible defence system that is interconnected by PTI and effector‐triggered immunity (ETI) (Jones & Dangl, 2006; Yuan, Jiang, et al., 2021; Yuan, Ngou, et al., 2021). Activation of this inducible system results in the synthesis of a large array of proteins including PR proteins. PR proteins are present in all plant species and function as core components of the inducible innate defence upon biotic and abiotic stress (van Loon et al., 2006). These PR proteins were initially numbered according to their molecular mass upon SDS‐PAGE and have subsequently been classified into 17 confirmed families, with PR1 proteins, 14‐kDa secreted glycoproteins, being the smallest. Most members of the PR protein family have been assigned specific biochemical functions such as proteinase inhibitors (PR6), plant defensins (PR12), ribonucleases (PR10), chitinases (PR3), β‐1,3‐glucanases (PR2), thaumatin‐like proteins (TLPs, PR5), peroxidases (PR9), thionins (PR13), and lipid transfer proteins (LTPs, PR14). Thanks to their enzymatic activity, they as a direct line of defence against fungal and bacterial pathogens (van Loon et al., 2006; van Loon & van Strien, 1999). However, the biochemical activity of PR1 remains an open question in the research community.

PR1 proteins, one of the three founding members of the CAP protein superfamily, are ubiquitous and have been identified in many different plant species (Fraser, 1981; Kothari et al., 2016; Lawrence et al., 1996; Shin et al., 2014; van Loon et al., 2006). They are among the most strongly expressed and abundant PR proteins and can account for up to 2% of total leaf proteins in tobacco (Lu et al., 2011; Niderman et al., 1995; van Loon & van Kammen, 1970; van Loon & van Strien, 1999). Some PR1 family members from tomato, potato, oilseed rape, and maize have IgE‐sensitizing capacity and thus act as plant allergens (Wangorsch et al., 2022). Most PR1 family members contain only a CAP domain and relatively short C‐ and N‐terminal extensions, indicating that the CAP domain determines their function in plant pathogen defence. Plants often produce dozens of different PR1 isoforms, which have been classified according to their isoelectric point as either acidic or alkaline (Dixon et al., 1991; Sessa et al., 1995). They are mostly secreted into the apoplastic space and predominantly localize adjacent to the site of lesion (Carr et al., 1987; Lincoln et al., 2018). The model plant *Arabidopsis thaliana*, for example, contains 21 PR1‐like proteins in addition to PR1 (AT2G14610) (Laird et al., 2004). However, only PR1 is transcriptionally induced upon pathogen attack. Expression of PR1 is controlled by the stress hormone salicylic acid (SA), and the rate of PR1 transcription is frequently used to monitor SA‐induced immune pathways and systemic acquired resistance (SAR) (Vlot et al., 2009).

Compared to their homologues in phytopathogens, the plant's own CAP proteins have been studied more extensively. An increasing body of evidence indicates that PR1 functions to mount an efficient immune response against pathogen attack, suggesting a role of both phytopathogen CAPs/VAPs and plant PR1 proteins as putative resistance‐breeding targets (Breen et al., 2017; Lincoln et al., 2018; Wangorsch et al., 2022). Here, we summarize more recent advances in characterizing PR1 function and their interacting partners, produced either by the host plant itself or by the invading phytopathogen (Table 2).

**TABLE 2:** Overview of pathogen effectors and plant proteins interacting with PR1.

Plant PR‐1 protein			Interactor			References
Host plant	Name	GenBank accession no.	Phytopathogen	Name	GenBank accession no.	
*Solanum tuberosum*	StPR1.2	XP_006367102.1	*Phytophthora infestans*	AMPK (PiAMPKα, PiSNF1, PiAMPKβ, PiAMPKγ)	PITG_07910, PITG_14707, PITG_14586, PITG_17395	Luo et al. (2023)
*Arabidopsis thaliana*	AtPR1	AT2G14610	*Sclerotinia sclerotiorum*	SsCP1	XP_001588549 (SS1G_10096)	Yang et al. (2018)
*Triticum aestivum*	TaPR1‐5	HQ541965	*Parastagonospora nodorum*	PnToxA	DQ423483	Lu et al. (2014)
	TaPR1‐1	HQ541961	*P. nodorum*	SnTox3	SNOG_08981	Breen et al. (2016); Sung et al. (2021)
	TaPR1a	FJ815169	*Puccinia striiformis* f. sp. *tritici*	PNPi	KT764125	Bi et al. (2020)
*Hordeum vulgare*	PR1a, PR1b	BAJ96307, X74940	*Blumeria graminis* f. sp. *hordei*	CSEP0055 (BghEfc3)	GT883397–GT883453	Godfrey et al. (2010); Zhang et al. (2012)
*Solanum lycopersicum*	SlPR1	P04284	*F. oxysporum* f. sp*. lycopersici*	FolSvp1	FOXG_11456	Li et al. (2022)
	P14a	P04284	–	Rac1 complex (HSP70, RACK1a, HSP90, SGT1)	Solyc09g010630.2.1, Solyc06g069010.2.1, Solyc07g065840.2.1, Solyc03g007670.2.1	Lincoln et al. (2018)
*T. aestivum*	TaPR1‐4	HQ848391/Q94f73	–	TaTLP1	KJ764822	Wang et al. (2020)
	TaPR1a	FJ815169	–	TaLTP3a	AY226580.1	Zhao et al. (2021)
	TaPR1‐5	HQ541965	–	TaPR1‐5	HQ541965	Lu et al. (2013)
*Triticum turgidum* subsp. *durum*	TdPR1.2	AB711115.1	–	TdCaM1.3	MW057248	Ghorbel et al. (2021)

–, interactor is from plant host.

### 4.1 Plant PR1 has strong antimicrobial activity

A strong antimicrobial activity of PR1 was first established in 1995, when zoospores of *Phytophthora infestans* were challenged with purified PR1 proteins from tobacco or tomato (Niderman et al., 1995). In these in vitro assays, PR1 reduced spore germination by more than 90%, and overexpression of PR1 in planta inhibited colonization by oomycetes on tomato and tobacco and of the rust fungus *Uromyces fabae* on broad bean (Alexander et al., 1993; Li et al., 2011; Niderman et al., 1995; Rauscher et al., 1999). After these landmark studies, continuous work further revealed the direct antimicrobial activity of PR1 proteins in vitro against different phytopathogens, including oomycetes, fungi, and bacteria (Gamir et al., 2017; Ghorbel et al., 2021; Guo et al., 2022). However, the mechanism underlying this antimicrobial activity of PR1 proteins remains to be characterized. The observation that transgenic plants overexpressing PR1 show increased resistance to biotic stress as well as abiotic challenges suggests not only that induction of PR1 serves to counter pathogens, but also that it is sufficient to induce stress resistance more generally (Kiba et al., 2007; Sarowar et al., 2005; Shin et al., 2014).

### 4.2 Plant PR1 is proteolytically cleaved to generate a signalling peptide

A systematic mass spectrometric approach to identify peptides that are induced by both wounding and methyl jasmonate treatment of tomato revealed a peptide derived from the 11 C‐terminal residues of PR1, termed CAP‐derived peptide 1 (CAPE1), harbouring a PxGNxxxxxPY signature sequence (Chen et al., 2014) (see Figure S1). The CAPE1 peptide is bioactive as it induces ROS formation when applied to tomato leaves. It also induces genes involved in the stress response, the innate immune response, defence against bacteria, and SAR. Plants treated with CAPE1 were resistant to *Pseudomonas syringae* and did not mount an HR (Chen et al., 2014). Even though HR was not induced to contain pathogen spread, CAPE1 was still sufficient to activate a defence response and limit bacterial colonization (Chen et al., 2014). Thus, CAPE1 may activate a novel damage‐associated molecular pattern (DAMP) signalling pathway to induce defence response genes and immunity.

Interestingly, the CAPE1 motif of PR1 is conserved across different flowering plants, ranging from monocots to dicots (Figure S1). In addition, residues preceding the putative cleavage site match a conserved CNYx motif, suggesting that the processing endopeptidase is functionally conserved as well. In agreement with a conserved signalling function of CAPE1, a corresponding C‐terminal peptide from *Arabidopsis* PR1 also has immune‐activating properties (Chen et al., 2014). Similarly, CAPE1 of wheat PR1 is sufficient to induce an immune response and to repress infection by *Parastagonospora nodorum* (Sung et al., 2021). Processing of PR1 and release of CAPE1 is likely to occur in the extracellular space, as treatment of the apoplastic fluid with serine‐protease inhibitors prevents the accumulation of the peptide (Sung et al., 2021).

Consistent with the observation that expression of PR1 in *Arabidopsis* is regulated by low temperature or high salt concentrations, levels of the CAPE1 are systemically enhanced upon salt stress (Chien et al., 2015; Seo et al., 2008). On the other hand, exogenous application of CAPE1 represses the induction of salt‐induced genes, and hence renders plants salt‐sensitive, indicating that CAPE1 signals both abiotic and biotic stress responses (Chen et al., 2014; Chien et al., 2015).

### 4.3 Plant PR1 is a key target of different fungal effector proteins

During plant–pathogen interactions, plants use plasma membrane‐localized pattern recognition receptors (PRRs), mainly receptor‐like kinases (RLKs) and receptor‐like proteins (RLPs), to recognize microbe‐associated molecular patterns (MAMPs)/PAMPs/DAMPs and to initiate a basal immune response, known as PAMP/MAMP/DAMP‐triggered immunity (Bigeard et al., 2015; Jones & Dangl, 2006). On the other hand, pathogens secrete effectors into the host's extracellular or intracellular space to restrain its PTI response or even to induce effector‐triggered susceptibility (Jones & Dangl, 2006).

Given the strong antimicrobial activity of PR1 and the immune signalling function of CAPE1, it is not surprising that pathogens have developed different strategies to counter PR1 function through effectors, either by blocking induction of PR1 expression or by interfering with its functions in the apoplastic space and thereby suppress the immune responses of the host.

#### 4.3.1 The necrotrophic pathogen effectors ToxA, Tox3, and the cerato‐platanin protein CP1 all target wheat PR1

ToxA is probably one of the best‐characterized effector proteins, which directly interacts with plant PR1. SnToxA is secreted by the causative agent of wheat tan spot disease, *Pyrenophora tritici‐repentis*, to trigger necrosis. Once inside the host cell, ToxA targets ToxABP1, a chloroplastic protein essential for photosynthesis, thus leading to cell death. ToxA may act both as an elicitor and as a virulence factor because a large number of defence‐related genes, including those for PR proteins, are upregulated in ToxA‐sensitive wheat cultivars (Pandelova et al., 2012). It has been proposed that ToxA, and perhaps other fungal effectors, may exploit plant defence mechanisms to induce host cell death for survival of the necrotrophic pathogens (Lai & Mengiste, 2013; Oliver et al., 2012). Interestingly, SnToxA specifically targets a particular homodimeric isoform of PR1, TaPR1‐5, and this site‐specific interaction is required for the function of SnToxA in promoting necrosis (Lu et al., 2014; Sung et al., 2021). Thus, binding of SnToxA to TaPR1‐5 appears to neutralize the immune‐protective function of wheat PR1‐5, potentially through CAPE1 signalling. However, the precise mode of action of this neutralizing activity of SnToxA remains to be determined. A second effector secreted by *P. nodorum*, SnTox3, interacts with a broader range of wheat PR1 members than SnToxA and inhibits release of CAPE1, thereby suppressing immune defence (Breen et al., 2016; Sung et al., 2021).

While exposure of wheat to SnToxA induces strong expression of TaPR1‐5 and this renders the host vulnerable to SnToxA‐induced necrosis (Lu et al., 2014), overexpression of PR1 in *Arabidopsis* renders the host resistant to a whole set of phytopathogens, including *Sclerotinia sclerotiorum*. One of the proteins that are important for the virulence of *S. sclerotiorum* is a member of the cerato‐platanin (CP) protein family. These are unusual cell wall‐associated proteins that are only present in filamentous fungi, but not restricted to phytopathogenic species. CP proteins are an interesting family of abundant small, self‐assembling, cysteine‐rich, secreted proteins with surfactant properties, and they have been proposed to modify the hydrophobicity of the fungal hydrosphere (Gao et al., 2020).

**FIGURE 2:**
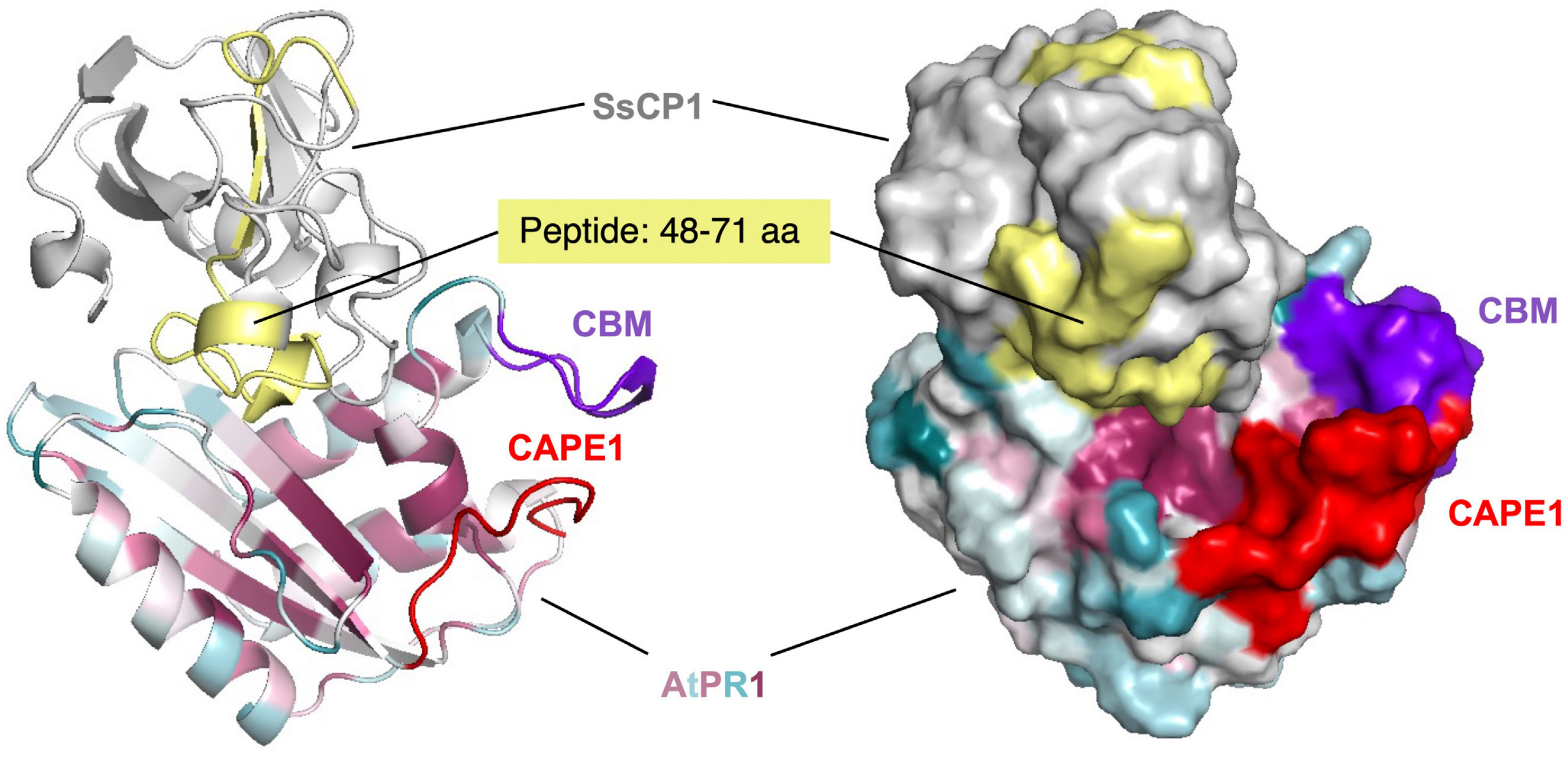
Docking of the CAP domain of AtPR1 with SsCP1. Protein docking was performed by UCSF Chimera (Pettersen et al., 2004). Conserved elements as determined by ConSurf (Ashkenazy et al., 2016) are indicated in red, and the CBM domain and CAPE1 motif are indicated. The SsCP1 peptide (amino acids 48–71) involved in binding with AtPR1 is shown in yellow. The structure is represented by a ribbon diagram and as a space‐filling model.

The CP protein from *S. sclerotiorum*, SsCP1, acts as a PAMP elicitor, thus inducing the expression of host defence genes and the production of ROS (Yang et al., 2018). SsCP1 triggers a local HR‐like cell death in plant leaves following surface application (Frías et al., 2013). Overexpression of SsCP1 in tobacco induces significant cell death and its overexpression in *Arabidopsis* results in an increased concentration of SA and higher levels of resistance against several pathogens (Yang et al., 2018). Interestingly, SsCP1 interacts with *Arabidopsis* PR1 in the apoplastic space in planta and promotes infection with *S. sclerotiorum*, whereas overexpression of PR1 enhances resistance (Yang et al., 2018). Direct interaction between AtPR1 and SsCP1 is consistent with our in silico docking experiments, revealing tight interaction between these two proteins (Figure 2). These observations have been taken to suggest that PR1 may serve as a possibly “universal” target of secreted pathogen effectors to impair the immune defence of the host (Yang et al., 2018).

#### 4.3.2 PR1 is the target of the NPR1 interactor from the biotrophic *Puccinia striiformis* and interacts with calmodulin

The function of PR1 as a target of secreted pathogen effectors is further underscored by its interaction with the conserved fungal effector *Puccinia* Nonexpressor of PR genes 1 (NPR1) interactor (PNPi) from the biotrophic wheat rust fungus *P. striiformis* and with the barley powdery mildew effector CSEP0055 (Bi et al., 2020; Zhang et al., 2012). Wheat lines overexpressing PNPi exhibit a reduced degree of SAR, possibly due to downregulation of TaPR1a and other PR family members (Bi et al., 2020). PNPi suppresses SAR by targeting NPR1, a key master regulator of SA signal transduction and transcriptional activation of PR‐related antimicrobial genes (Wang et al., 2016). Upon pathogen infection or exogenous SA application, NPR1 is translocated from the cytoplasm to the nucleus to induce expression of PR proteins (Backer et al., 2019; Kong et al., 2021). Overexpression of PNPi in barley cells downregulates the induction of PR genes after pathogen infection (Wang et al., 2016). However, PNPi does not only affect the function of NPR1, but it also physically interacts with TaPR1a in the apoplastic space (Bi et al., 2020). Unusual for a pathogen effector, PNPi seems to be conserved among a wide range of plant pathogens, suggesting that it plays an important role in suppression of the host immune response. Thus, PNPi acts as a negative regulator of the expression of PR family members through NPR1, but how this signal is exactly transmitted from the apoplastic space into the host cell's cytoplasm remains to be established.

It is interesting to note that many of the interactors of PR1, including Tox3 and PNPi, bind the protein at its C‐terminal part and thus may interfere with processing of CAPE1 (Bi et al., 2020; Breen et al., 2016; Sung et al., 2021). In addition, this part of the PR1 protein, connecting helix α4 with sheet β2, in both monocotyledonous and dicotyledonous plants harbours a calmodulin‐binding domain (Figure S1). Calmodulins are small acidic calcium‐binding proteins that decode Ca^2+^ signals to modulate an extensive range of cellular processes, including Ca^2+^ homeostasis, stress responses, and gene regulation (Carafoli & Krebs, 2016). PR1 interacts with calmodulins in a Ca^2+^‐dependent manner. The antimicrobial activity of PR1 proteins in vitro is stimulated by Ca^2+^/calmodulin and greatly decreases when the conserved calmodulin‐binding site is deleted from the C‐terminal part of PR1 (Ghorbel et al., 2021). These observations raise the possibility that the Ca^2+^/calmodulin complex functions in the regulation of plant PR1 during cellular responses to external signals (Ghorbel et al., 2021). Given that the calmodulin‐binding site of PR1 is located between the CAP domain and a proteolytic cleavage signal that releases a signalling peptide, CAPE1, from PR1 (see below, Figure S1), it seems possible that processing of this signalling peptide is regulated by Ca^2+^/calmodulin or by one of the many calmodulin‐like proteins present in plants (Virdi et al., 2015).

#### 4.3.3 Translocation of PR1, a way to prevent generation of the CAPE1 signalling peptide

Accumulating evidence indicates that translocation of PR1 from the apoplastic space, where the CAPE1 immune signalling peptide is generated and released, into the host cell may be one strategy to neutralize the antimicrobial action of PR1 and prevent the release of the CAPE1 peptide. One effector that may function this way is the secreted virulence‐related protein 1 (FolSvp1) from *F. oxysporum* f. sp. *lycopersici* (Fol). FolSvp1 directly interacts with PR1 and relocates PR1 from the apoplast to the host nucleus (Li et al., 2022). This translocation abolishes the generation of the CAPE1 signalling peptide, thereby impairing the plant immune response and resulting in successful invasion of tomato host plants. Thus, FolSvp1 hijacks apoplastic PR1 and translocates it into the host nucleus, and this translocation is dependent on the nuclear localization signal (NLS) present in FolSvp1. Substitution of the signal sequence of PR1 by an NLS results in nuclear localization of NLS‐PR1. Under these conditions, the CAPE1 peptide cannot be generated and plants overexpressing nuclear‐localized PR1 do not display increased resistance against pathogens (Li et al., 2022). On the other hand, potato PR1 has been reported to be translocated into oomycetes and to inhibit their growth by targeting subunits of the AMP‐activated protein kinase complex, suggesting a possible cross‐kingdom translocation of secreted PR1 (Luo et al., 2023).

### 4.4 Plant PR1 recruits additional PR proteins, including thaumatin‐like proteins (PR5) and lipid transfer proteins (PR14), to increase pathogen resistance

The sweet‐tasting thaumatin‐like proteins (TLPs), which include osmotin and zeamatin, belong to the PR5 protein family. TaTLP1 was identified in wheat in response to infection with *Puccinia triticina* (Hakim et al., 2018). TLPs are secreted into the apoplastic space, where they rapidly accumulate to high levels in response to biotic or abiotic stress, and they exhibit antifungal activity in various plant species. Gene silencing and pathology tests indicate that TaTLP1 is involved in resistance to the wheat leaf rust pathogen (Zhang, Wang, et al., 2018). Interestingly, TaTLP1 interacts with TaPR1‐4, and in vitro, the antimicrobial activity of the purified heteromeric complex against *Escherichia coli* is higher than that of either TaTLP1 or TaPR1‐4 alone. Furthermore, cosilencing of both genes significantly reduces the accumulation of ROS and enhances susceptibility to leaf rust, indicating that these two proteins cooperate to protect wheat against biotic stress in a ROS‐dependent manner (Wang et al., 2020). Resistance activity of PR1 maps once again to its C‐terminal part. The CAPE1 peptide alone, when infiltrated into wheat leaves, increased resistance and enhanced the defence response induced by TaTLP1 (Wang et al., 2022).

In addition to directly interacting with TLPs (PR5), PR1 also interacts with PR14 proteins, which are members of the lipid transfer protein (LTP) family. These nonspecific LTPs bind to and transfer lipids between membranes in vitro, and they may act as lipid sensors or sequester lipids to dampen their potential signalling function in vivo (Missaoui et al., 2022). The antimicrobial action of wheat PR1 against *P. triticina* is enhanced by the presence of TaLTP3 in the apoplastic space (Zhao et al., 2021). Overexpression of TaLTP3 induces transcription of *TaPR1a* and activates multiple plant hormone pathways, including the SA, jasmonic acid, and auxin pathways, and results in enhanced resistance to the leaf rust pathogen. Interestingly, TaLTP3 physically associates with TaPR1a in the apoplast and plants overexpressing both proteins display enhanced production of ROS during the defence response. Thus, the interaction between PR1 and TaLTP3 enhances the antimicrobial activity of PR1, pointing to a possible function of PR1 in LTP‐dependent lipid sequestration (see below). LTPs belonging to the PR14 family constitute a large protein family that is present in all land plants, and they have been implicated in various processes including defence against biotic and abiotic stress (Salminen et al., 2016). For example, overexpression of wheat TaLTP5 results in increased pathogen resistance and when tested in vitro, some of these LTPs exhibit antimicrobial activity (Maldonado et al., 2002; McLaughlin et al., 2020; Sun et al., 2008; Zhu et al., 2012).

The hexaploid wheat genome encodes a total of 330 LTPs, many of which are highly induced by abiotic stresses such as drought or salinity (Fang et al., 2020). These secreted nonspecific LTPs thus share some of the key properties of PR1 family members and physically interact with PR1 to increase pathogen resistance in planta. The association and complex formation of PR1 with PR5 or PR14 family members in the apoplastic space increases the antimicrobial activities of these PR proteins in a ROS‐dependent manner. Future studies focusing on dissecting the synergistic functions between PR5 (TLPs), PR14 (LTPs), and PR1 family members are expected to shed some light on the synergistic mode of action of these proteins either in directly attacking the microbial invader or in the ROS‐mediated defence pathway.

## 5 CAP PROTEINS BIND LIPIDS AND LIPIDS HAVE IMMUNOMODULATORY ACTIVITIES

Lipids are major constituents of prokaryotic and eukaryotic membranes and have diverse biological functions in energy storage, signal transduction, and stress responses. Several independent observations indicate that CAP family members from different organisms, including fungi, nematodes, plants, and humans, can bind lipids, particularly eicosanoids, fatty acids, sterols, phosphatidylinositol, and negatively charged phospholipids (Choudhary & Schneiter, 2012; Darwiche, Mène‐Saffrané, et al., 2017; Van Galen et al., 2010; Xu et al., 2012).

During plant–pathogen interactions, membranes establish an interface between the two organisms, and membrane lipids and lipid‐derived messengers serve as signals in plant–pathogen communication. Thus, lipid‐mediated cross‐kingdom communication between host and pathogen is a rapidly emerging field in molecular plant–fungal interactions. Amidst the cross‐talk between host plants and fungal pathogens lies a distinct class of oxygenated derivatives of polyunsaturated fatty acids termed oxylipins (Christensen & Kolomiets, 2011). Fungal oxylipins are important for successful colonization of the host, division, and synthesis of toxins, while plant oxylipins, such as jasmonic acid and related jasmonate metabolites, function in reproduction and development and facilitate resistance to pathogen attack (Siebers et al., 2016). However, whether CAP family proteins and particularly plant PR1 can bind these oxylipins and thereby potentially dampen their signalling function remains to be tested. Alternatively, as discussed for sterols below, binding of such oxylipins at the fatty acid binding pocket of CAP proteins may affect the conformation of the C‐terminal part of the proteins and thereby potential recognition by a peptidase and hence impact CAPE1 release and its immune signalling function.

Another potential connection between PR1 function and lipids is given by the observation that overexpression of PR1 proteins in tomato roots suppresses a programmed cell death response induced by the mycotoxin fumonisin B1 (Lincoln et al., 2018). Fumonisin B1 is a lipid‐like toxin that is produced by several species of *Fusarium* moulds and it specifically inhibits the synthesis of ceramide, a precursor lipid for the production of more mature sphingolipids, including galactosyl ceramide and inositol phosphoryl ceramides (Mamode Cassim et al., 2020). This anti‐apoptotic activity of PR1 is mediated by modulating levels of ROS and is conserved across kingdoms, as the expression of a human CAP family member, glioma pathogenesis‐related protein 1 (GLIPR1), or of a dog hookworm PR1 orthologue in transgenic tomato roots also protects plants against fumonisin B1 toxicity (Lincoln et al., 2018). Given that CAP proteins bind lipids, it seems possible that they could neutralize the inhibitory action of fumonisin B1, which itself has lipid‐like properties, by binding and sequestering it in the apoplastic space. This would prevent the toxin from reaching its target protein, which is localized in the endoplasmic reticulum. On the other hand, pulldown assays identified members of the Rac1 immune complex as putative interactors of the tomato PR1 orthologue P14a, which has been taken to suggest that suppression of cell death by PR1 may be mediated through modulating Rac1‐mediated innate immunity (Kawasaki et al., 1999; Lincoln et al., 2018). Rac1 is localized at the plasma membrane and upon activation by an elicitor such as chitin, it transiently associates with lipid microdomains enriched in sterols and sphingolipids (Fujiwara et al., 2009; Nagano et al., 2016). Blocking ceramide synthesis by fumonisin B1 affects the formation and function of raft‐dependent signals and impairs Rac1 activation (Lingwood & Simons, 2010; Song et al., 2021). Whether PR1 family members associate with or affect the formation of plasma membrane microdomains has not yet been investigated but this may explain the interaction with Rac1. As observed with fumonisin B1‐treated plant cells, mammalian CAP family members also exert an anti‐apoptotic function, and this may explain their inhibitory activity on tumour progression (Li et al., 2013).

## 6 OOMYCETES ARE STEROL AUXOTROPHS AND SENSITIVE TO THE STEROL‐BINDING ACTIVITY OF PR1

**FIGURE 3:**
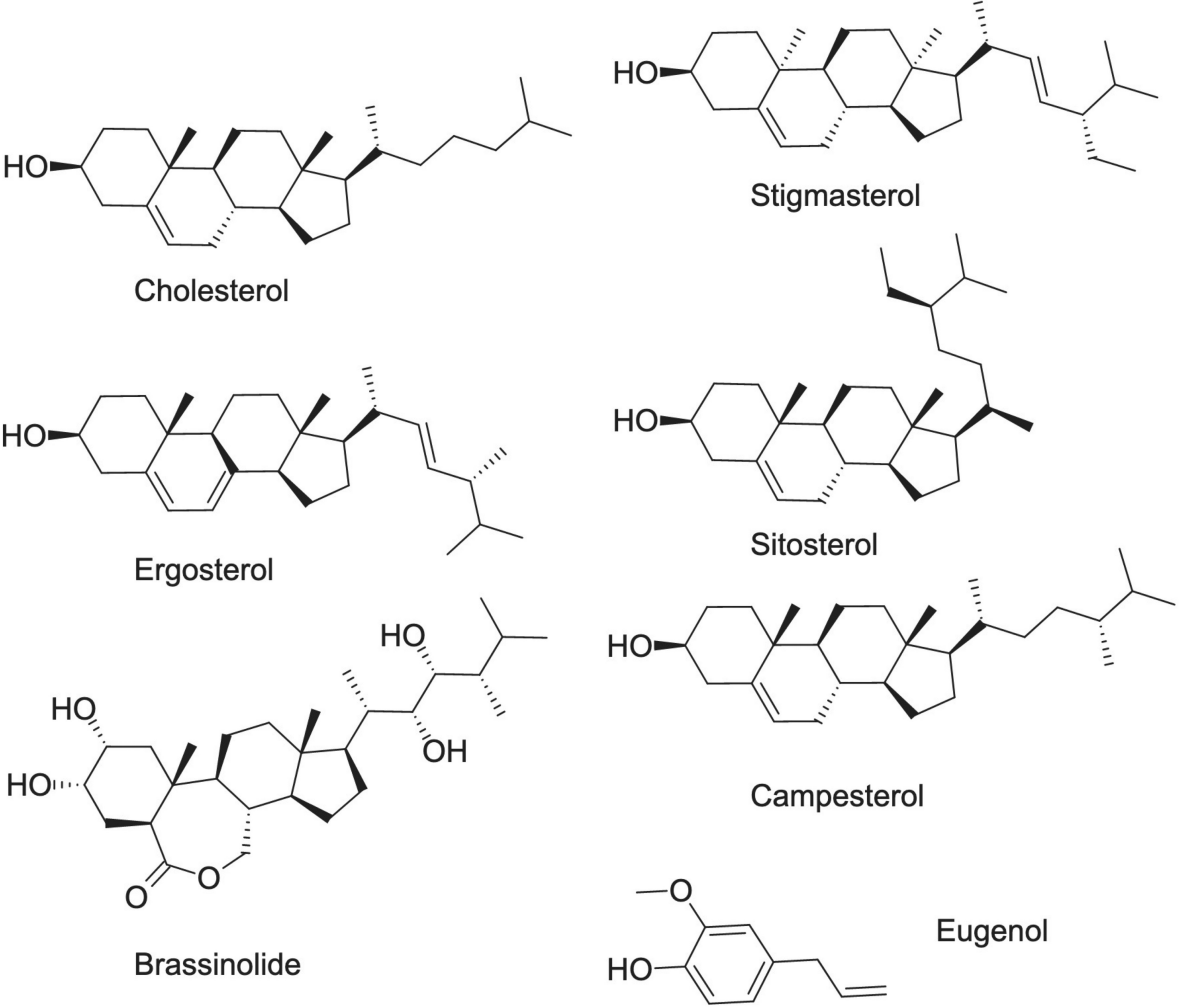
Comparison of sterol structures between fungi, mammals, and plants. The structural properties of the fungal ergosterol, mammalian cholesterol, the β‐phytosterols stigmasterol, sitosterol, and campesterol, and the plant signalling steroid brassinolide are shown. Note that the three phytosterols share the same tetracyclic ring structure with the mammalian cholesterol but differ in their aliphatic side chains. Also shown is the structure of eugenol, an allylbenzene class of aliphatic compounds that can also be bound by PR1 proteins.

Sterols constitute a major class of lipids found in membranes, particularly the plasma membrane, of all eukaryotes. While mammals synthesize cholesterol as their major sterol, fungal cells make ergosterol and plants typically make a mixture of three different β‐phytosterols, sitosterol, stigmasterol, and campesterol. These sterols share a similar tetracyclic ring structure but differ slightly in the structure of their aliphatic side chains (Daum et al., 1998; Hartmann, 1998) (Figure 3). Importantly, the fungal ergosterol is not made in plants and is recognized as “nonself” (Weete et al., 2010). Thus, in addition to the fungal cell wall components chitin, chitosan, and β‐glucans, ergosterol also acts as a fungal MAMP to induce a PTI response (Klemptner et al., 2014). Induction of the PTI response by ergosterol is highly specific, as it is not observed when leaves are treated with mammalian cholesterol (Granado et al., 1995; Lochman & Mikes, 2006; Vatsa et al., 2011). The precise mechanism of perception of the fungal sterol ergosterol as “nonself” by plants remains to be elucidated. It has been proposed that plants possess ergosterol receptors or that the sequestration of ergosterol from fungal membranes into the plasma membrane of the host plant results in perturbations of membrane microdomains, which in turn affects signal transduction pathways that are initiated at these membrane domains (Khoza et al., 2019; Rossard et al., 2010; Xu et al., 2001).

The complex mixture of sterols made by plants modulates the function of many different membrane‐associated processes, including membrane transport, protein trafficking, the formation of membrane microdomains, signal transduction, and biotic and abiotic stress responses (Griebel & Zeier, 2010; Malinsky et al., 2013; Sharfman et al., 2014; Wang et al., 2012). In addition to the bulk function of phytosterols in modulating membrane properties, these sterols can be oxidized to signalling lipids, such as brassinosteroids, which function during growth and development but also serve to counteract abiotic and biotic stress (Nolan et al., 2020). Brassinosteroids are perceived by binding to BRI1, a leucine‐rich repeat (LRR) domain‐containing receptor‐like kinase (LRR‐RLK). Whether these receptors could also detect more hydrophobic membrane‐embedded sterols such as the fungal ergosterol has to our knowledge not been tested. However, an LRR‐RLK has been identified as a potential ergosterol‐responsive protein (Khoza et al., 2019), but given the low solubility of ergosterol compared to steroids, ligand solubility would probably impose restrictions on its perception by surface‐bound LRR‐RLKs.

It is interesting to note that oomycetes abundantly produce and secrete a family of small proteins known as elicitins, which constitute a well‐characterized class of MAMPs and induce a strong HR in several plants, including tobacco (Derevnina et al., 2016). Elicitins share some structural similarity with nonspecific plant LTPs, and the purified protein binds sterols or free fatty acids and promotes sterol transfer between artificial membranes in vitro (Blein et al., 2002; Derevnina et al., 2016; Mikes et al., 1997; Osman et al., 2001). On the other hand, the LTPs made by plants mostly bind flexible linear lipids such as acyl chains or lyso‐phospholipids rather than lipids containing rigid polycyclic structures as is the case with sterols (Salminen et al., 2016). The fact that most oomycetes cannot synthesize their own sterols for their growth and reproduction and hence are sterol auxotrophs led to the proposition that oomycetes secrete elicitins to bind and sequester sterols from the host plant and shuttle these sterols back to meet their own demand (Dahlin et al., 2017; Gonzales & Parks, 1981; Ponchet et al., 1999).

Sequestering sterols from the host's plasma membrane by elicitin increases plasma membrane fluidity and induces ROS production (Sandor et al., 2016). By contrast, treatment of wheat roots with methyl‐β‐cyclodextrin, a sterol‐sequestering low‐molecular‐weight compound, is not sufficient to induce oxidative stress, unless it is accompanied by an enhanced ion permeability of the plasma membrane (Valitova et al., 2014). Thus, further work is needed to more precisely define the role of plasma membrane sterols and the function of sterol‐dependent signalling microdomains as mediators of an immune response, as well as to understand how extracellular sterol‐sequestering proteins, such as elicitins and PR1 or PR14 proteins, could interfere with immune activation (Gronnier et al., 2018; Levental et al., 2020).

The immune‐activating function of elicitins might be mediated by the elicitin–sterol complex possibly through high‐affinity interactions with surface receptors, including the protein elicitin response (ELR) in potato, which signals through SERK3/BAK1 to mediate immunity (Du et al., 2015; Osman et al., 2001). Interestingly, brassinosteroids also signal through SERK3/BAK1, and activation of this signalling pathway is required to confer basal resistance of tobacco against *P. infestans* (Chaparro‐Garcia et al., 2011; Noman et al., 2020). However, whether elicitins bind brassinosteroids or related steroids and thereby could interfere with signalling remains to be tested. As is the case in mammalian cells, plant immune and growth receptors share common signalling components but their activation can result in vastly different responses, an enigma that has been attributed to distinct localization and activation of these receptors (Bücherl et al., 2017).

Proteomic profiling of the secretome of *P. infestans* revealed five CAP/SCP family members, suggesting that the secretion of elicitin does not simply substitute for CAP function, but these two protein families exert different, possibly complementary functions (Meijer et al., 2014). In addition, more recent observations indicate that sterol binding by elicitins is not required to elicit a PTI response, suggesting that the protein itself is recognized rather than the elicitin–sterol complex (Dokládal et al., 2012; Starý et al., 2019).

Importantly, growth of sterol auxotrophic phytopathogens such as the oomycete *Phytophthora* is particularly sensitive to PR1, whereas sterol prototrophs become highly sensitive only when their sterol synthesis is inhibited (Gamir et al., 2017). This growth inhibition by PR1 depends on the sterol‐binding function of the protein, as mutations that inhibit lipid binding render the protein nongrowth‐inhibitory (Gamir et al., 2017). In addition, this growth‐inhibitory activity of PR1 could be neutralized by addition of free sterols to the medium, indicating that the antimicrobial activity of PR1 is provided by its sterol‐binding capability. The fact that PR1 particularly inhibits sterol auxotrophic pathogens may explain why plants are themselves resistant. Binding of sterols by CAP proteins is not specific to a particular type of sterol, as these proteins can bind mammalian cholesterol, the fungal ergosterol, or the plant sitosterol (Choudhary & Schneiter, 2012; Darwiche & Schneiter, 2016). In addition, these proteins can also bind other small hydrophobic compounds such as eugenol. Eugenol is a member of the allylbenzene class of compounds that is present in clove oil, nutmeg, cinnamon, and bay leaf and is used as local antiseptic and anaesthetic (Barnes et al., 2007) (Figure 3). CAP proteins can bind eugenol in vitro, and overexpression of these proteins renders yeast cells resistant to eugenol toxicity (Choudhary & Schneiter, 2012; Darwiche & Schneiter, 2016). Depending on the precise sequence and structure of the flexible loop required for binding of sterols by CAP proteins, their specificity towards such small hydrophobic ligands may be subject to modulation and optimization (Choudhary et al., 2014).

**FIGURE 4:**
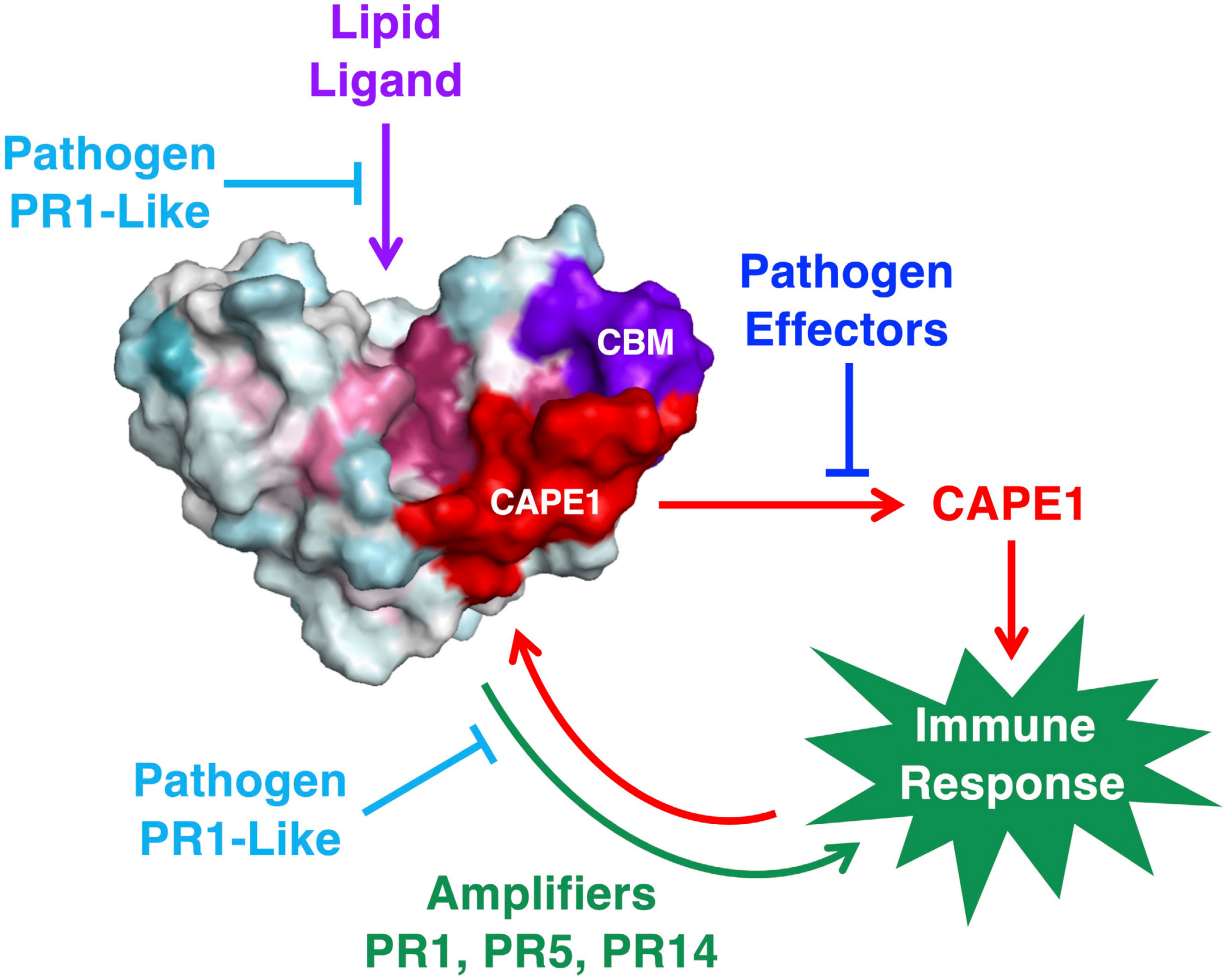
Possible model of PR1 function in the apoplastic space. In this schematic model, the PR1 protein, shown as a space‐filling model, perceives the presence of small lipid ligands such as sterols or oxylipids in the apoplastic space to facilitate release of the C‐terminal CAPE1 signalling peptide. Binding of PR1 by various pathogen effectors blocks the release of CAPE1 and hence suppresses the host's immune response. Once released, CAPE1 induces plant immunity and upregulation of PR proteins, particularly PR1, PR5, and PR14. PR1 can then interact with PR5 and/or PR14 in the extracellular space to amplify immunity by increasing the antimicrobial activity of the PR1 protein complex itself and/or through the release of more CAPE1. PR1‐like proteins secreted by various pathogens may either sequester the lipidic ligand to suppress perception of the initial signal or sequester the interactors of PR1 such as PR1 itself, PR5, and/or PR14.

While the previously mentioned results are consistent with a possible direct antimicrobial action of PR1 through the sequestration of sterols from the pathogen membrane, they do not exclude a more subtle action of PR1 on immune signalling of either the host or the pathogen (Han & Kahmann, 2019; Kazan & Gardiner, 2017). A sterol‐dependent activation of the host's immune system could, for example, be triggered through the release of the CAPE1 peptide. Interestingly, the CAPE1 C‐terminal part of PR1 is in close proximity to the flexible loop harbouring the CBM and it has been proposed that sterol binding could be coupled to the cleavage and release of the CAPE1 peptide (Breen et al., 2017) (Figures 1, 2, and 4). A possible model summarizing the interactions of PR1 with lipids, effectors, and the plant's own enhancers, such as PR5 and PR14, as well as their integration with CAPE1 signalling is proposed in Figure 4.

## 7 UNDERSTANDING THE MODE OF ACTION OF CAP PROTEINS DURING PLANT–PATHOGEN INTERACTION, ARE WE BEGINNING TO LOOK AT LIPIDS AND OTHER SMALL LIGANDS MORE CLOSELY?

Plants are under constant threat from phytopathogens. To develop sustainable disease prevention and control strategies, it is important to understand the molecular mechanisms that govern plant–pathogen interactions and to identify key virulence and resistance genes. Considering their wide participation in plant defence responses and pathogen virulence, PR1 proteins from both host plants and phytopathogens are among the prime targets. Lipids are major constituents of prokaryotic and eukaryotic membranes, but also serve important signalling functions, particularly in modulating stress and immune responses. During plant–pathogen interactions, membranes establish an interface between the two organisms, and membrane lipids and lipid‐derived messengers serve as signals in plant–pathogen communication. Given the recent identification of interaction partners of PR1 in the apoplastic space, including nonspecific LTPs (PR14), thaumatin/osmotin‐like proteins (PR5), and proteins such as SnTox3, which inhibit processing of PR1 and release of the immune stimulatory CAPE1 peptide, it could prove fruitful to focus more intensely on the role of lipid mediators in this cross‐kingdom interaction.

## Supporting information


**Figure S1.** Conservation of the CAPE1 peptide in plant PR1 proteins. Alignment of plant PR1 sequences shown in Table 2. The CAP1–4 signature motifs, the caveolin‐binding motif (CBM), the conserved histidine and glutamic acid residues, and cysteines are highlighted as shown in Figure 1. The calmodulin‐binding motif (CaMBD) is indicated in the green box. The CAPE1 cleavage site (CNYx) is indicated and the CAPE1 peptide is boxed in red.

## Data Availability

Data sharing is not applicable to this article as no new data were created or analysed.
