# Supplementary material for: The function of plant PR1 and other members of the CAP protein superfamily in plant–pathogen interactions
Source: Mol Plant Pathol. 2023 Mar 17;24(6):651–68. doi: 10.1111/mpp.13320 (PMC10189770; doi:10.1111/mpp.13320)

Supplementary Figure

Figure S1. Conservation of the CAPE1 peptide in plant PR1 proteins. Alignment of plant PR1 sequences shown in Table 2. The CAP1-4 signature motifs, CBM, the conserved histidine and glutamic acid residues and cysteines are highlighted as shown in Figure 1. The calmodulin binding motif (CaMBD) is indicated in the green box. The CAPE1 cleavage site (CNYx) is indicated and the CAPE1 peptide is boxed in red.

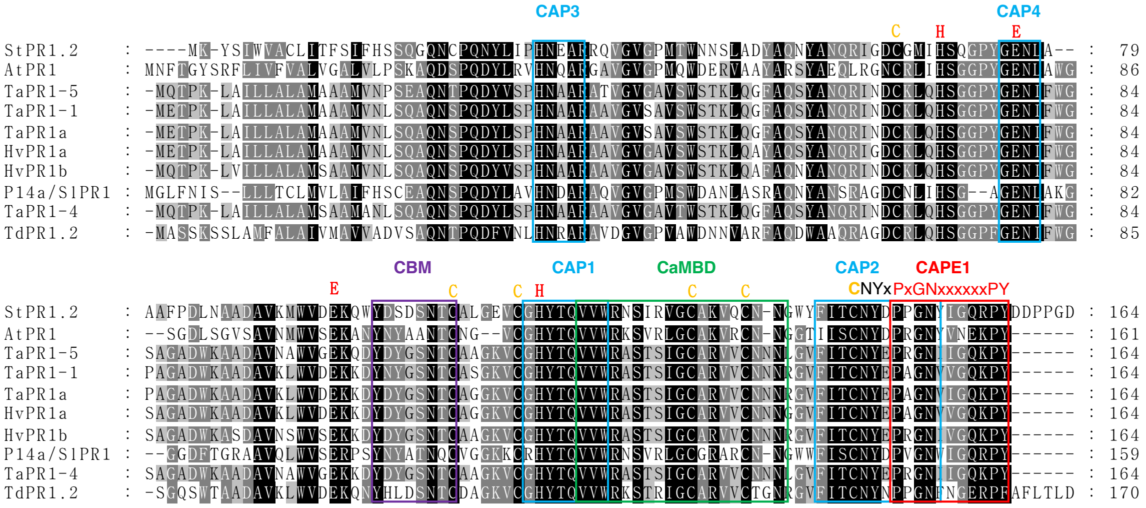

Supplement: Supplementary file 1 — Figure S1. Conservation of the CAPE1 peptide in plant PR1 proteins. Alignment of plant PR1 sequences shown in Table 2. The CAP1–4 signature motifs, the caveolin‐binding motif (CBM), the conserved histidine and glutamic acid residues, and cysteines are highlighted as shown in Figure 1. The calmodulin‐binding motif (CaMBD) is indicated in the green box. The CAPE1 cleavage site (CNYx) is indicated and the CAPE1 peptide is boxed in red. [file MPP-24-651-s001.pdf]
